# Linda’s Cars versus Dominik’s Dolls: How Do Pedagogical Educators in Training React to Children’s Violations of Gender Stereotypes?

**DOI:** 10.1007/s10508-024-02958-7

**Published:** 2024-08-06

**Authors:** Hannah Streck, Ursula Kessels

**Affiliations:** Department of Education and Psychology, Freie Universität Berlin, Habelschwerdter Allee 45, 14195 Berlin, Germany

**Keywords:** Gender nonconformity, Gender stereotypes, Gender socialization, Children, Toys

## Abstract

The gender stereotypes adults hold can influence whether they approve or disapprove of behavior shown by children, depending on whether this behavior is in line with stereotypes. Adults report negative evaluations toward children whose behavior does not adhere to gender stereotypes, particularly toward feminine boys. Whether pedagogical educators in training show negative reactions toward children who violate gender stereotypes has not been examined. We investigate this question by firstly assessing what gender stereotypes adults hold about children in Germany. In Study 1, we assessed descriptive, prescriptive, and proscriptive gender stereotypes identified by adults for children in German society. Stereotypes gathered from this first study were used to construct four vignettes of stereotypical and nonstereotypical boys and girls in order to examine how pedagogical educators in training (*N* = 414) evaluated these children in Study 2. We investigated ratings of one of these vignettes (2 × 2 between-participants design) regarding liking, perceived competence, creativity, self-esteem, prosocial behavior, as well as internalizing and externalizing problems. A series of ANOVAs revealed that girls displaying masculine behavior received advantageous ratings on competence, creativity, and self-esteem, while boys showing femininity were perceived as the most prosocial. More than gender nonconformity, masculinity and femininity strongly related to externalizing and internalizing problems, respectively. We review how our results in Germany differ from the literature originating in the USA, as we did not find backlash for feminine boys. Possible bias against femininity and toward masculinity within society and cultural and sampling factors is discussed.

## Introduction

### Social Cognitive Theory: Relevance of Stereotypes for Children

As they develop over childhood, children become more understanding of the complex phenomenon of gender. In theoretical frameworks, the role of social interactions and feedback is prominently featured (see Coyle & Fulcher, 2022). Social cognitive theory (Bussey & Bandura, 1999) highlights that an individual’s environment plays a major role in how gender is understood and enacted. From birth, children are exposed to three social modes of influence: modeling, enactive experience, and direct tuition (Bussey & Bandura, 1999). The relevance of gender stereotypes held by adult socializers is particularly pronounced in enactive experience and direct tuition: Enactive experience refers to how children display certain behavior and use cues of approval or disapproval by others to guide their future behavior. Direct tuition encompasses direct, verbal feedback from others about the appropriateness of one’s gendered behavior. A boy playing with dolls may note heads shaking or frowning and interpret that this behavior, which, when enacted by his sister, was met with encouragement, is not appropriate for himself on the basis of his gender (Bussey, 2011).

### Gender Stereotypes

Gender stereotypes have been defined as culturally shared characteristics ascribed to men and women based on their gender (Myers et al., 2010). Stereotypes about men and women fall into the categories agency and communion (Bakan, 1966), respectively. Agency refers to ambition, independence, and self-determination (Abele & Wojciszke, 2013) and is closely related to competence (Cuddy et al., 2008), instrumentality (Spence et al., 1974), and masculinity (Bem, 1981a). Communion, on the other hand, refers to an orientation toward other people, caring, and kindness (Abele & Wojciszke, 2013) and relates to warmth (Cuddy et al., 2008), expressiveness (Spence et al., 1974), and femininity (Bem, 1981a). Stereotypes, however, not only reflect traits but also appearance and behaviors deemed gender-appropriate.

### Descriptive, Prescriptive, and Proscriptive Gender Stereotypes

Researchers have posited that all stereotypes could be considered “descriptive” stereotypes, meaning that they describe how members of stereotyped groups typically behave (Koenig, 2018; Prentice & Carranza, 2002; Rudman & Glick, 2010; Sczesny et al., 2019). A subset of these stereotypes, “prescriptive” stereotypes, contain information about how each gender should behave (Prentice & Carranza, 2002; Rudman & Glick, 2010). “Proscriptive” gender stereotypes, a further subset of prescriptive stereotypes, contain information about how each gender should not behave (Rudman & Glick, 2010). Descriptive stereotypes serve as a cognitive simplification tool, allowing us to save time and energy when confronted with stereotyped group members (Eckes, 2008; Rudman & Glick, 2010). Prescriptive and proscriptive stereotypes, however, originate in system justification, where their function as guidance for behavior ensures that group members adhere to acceptable norms (Eckes, 2008; Rudman & Glick, 2010).

### Gender Stereotypes About Children

Investigations into the content of stereotypes have focused mainly on adults, though there are findings showing gendered stereotypes of children (Koenig, 2018; Martin, 1995; Sullivan et al., 2018, 2022; Zucker, 1977). Generally, stereotypes about children relate to their appearance and activities rather than their traits. Girls were described as (enjoying) playing with dolls and boys as (enjoying) playing with trucks (Koenig, 2018; Sullivan et al., 2018, 2022). Appearing masculine and feminine and playing with masculine and feminine toys were also prescriptive stereotypes for boys and girls, respectively (Koenig, 2018; Sullivan et al., 2018). Proscriptive stereotypes for boys related to appearing feminine and engaging with feminine activities (Koenig, 2018) and being dirty or appearing masculine for girls (Sullivan et al., 2022).

### Reactions to Violations of Gender Stereotypes

A wide variety of investigations showed stereotype violations are punished by others, with most research focusing on adults (Moss-Racusin et al., 2010; Nauts, 2015; see Rudman et al., 2012a, b; Rudman & Glick, 2001; Sanborn-Overby & Powlishta, 2020). Adolescents (Braun & Davidson, 2017; Young & Sweeting, 2004) and children (Blakemore, 2003; Kwan et al., 2020; Xiao et al., 2019; Zucker et al., 1995) also experience negative consequences for counterstereotypical behavior from their peers.

### Reactions to Violations of Stereotypes by Children

A number of studies showed that adults reacted more negatively to children who defied gender stereotypes than to children who adhered to stereotypes (Cahill & Adams, 1997; Coyle et al., 2016; Fagot, 1977; Feinman, 1981; Martin, 1990; Sandnabba & Ahlberg, 1999; Sullivan et al., 2018; Thomas & Blakemore, 2013). Some of these studies were conducted in the 1980s and 1990s, and social values can shift over these timeframes (Eagly et al., 2020). In the following sections, we will thus focus more heavily on more recent research.

#### Likability

The perceived likability of children was claimed to be a “primary measure of interest” (Sullivan et al., 2018) in one study from the USA, which found children described as atypical to be liked less than their typical peers by adults. Girls described as behaving stereotypically feminine were rated as most likable, significantly more so than masculine girls. A different vignette study also found the typical girl, characterized as a girl showing positive femininity, to be the most likable. “Tomboys,” girls enacting positive masculinity, were ambivalently evaluated (Coyle et al., 2016). Ratings of likability of typical and nontypical boys revealed boys enacting femininity to be rated as the least likable (Sullivan et al., 2018). “Sissies,” boys enacting negative femininity, were similarly not well-liked (Coyle et al., 2016). Boys showing positive femininity (“mama’s boys”), however, were more tolerated by adults.

A more specific sample of adults, preschool teachers, had more lenient attitudes toward girls showing nonconformity but reacted more negatively toward nonconforming boys (Cahill & Adams, 1997). Boys engaging in feminine play also received more criticism, from teachers (Fagot, 1977). However, in some work teachers reported more positive evaluations toward feminine boys compared to masculine boys (Piché & Plante, 1991).

#### Competence

Past research from the USA has investigated the perceived competence of gender-typical and atypical children (Coyle et al., 2016). Adults rated a hypothetical typical girl as the most competent but also indicated finding a girl described as enacting positive masculinity rather competent. Similarly, target boys enacting positive femininity were rated as rather competent, more so than the typical boy, but “sissies” were found to be the least competent (Coyle et al., 2016). The boys and girls described as showing positive femininity and masculinity, respectively, were not penalized in terms of competence, but a boy displaying negative femininity was. These findings support linking perceived competence with gender typicality but also suggest that some gender atypicality can have positive associations with competence ratings. Furthermore, a study with adolescents showed targets described as gender atypical to be perceived as more competent than gender stereotypical targets, this effect being significant in participants with high SES (Meimoun et al., 2024).

#### Experiencing Problems

Studies have measured the extent to which adults believed gender nonconforming children experience problems in their happiness, relationships with others (Coyle et al., 2016), and psychological adjustment (Thomas & Blakemore, 2013). Internalizing tendencies can encompass emotional problems and social isolation from peers according to the Strength and Difficulties Questionnaire, an established measure of adjustment (Goodman, 2001; Goodman et al., 2010). Externalizing problems may relate to hyperactivity or behavioral conduct issues (Goodman, 2001; Goodman et al., 2010). Past researchers constructed scales of externalizing and internalizing tendencies, encompassing aggression, misconduct and self-esteem, worrying, respectively. They revealed it was not nonconformity itself but the typing of behavior as masculine and feminine that predicted these latter ascribed pathologies (Thomas & Blakemore, 2013). Masculine behavior was related to whether children were believed to display externalizing tendencies, while feminine behavior was related to internalizing tendencies, irrespective of child gender (Thomas & Blakemore, 2013).

#### Differential Reactions to Children’s Stereotype Violations by Gender

A persistent theme in this literature is the comparatively more positive attitudes toward nonconforming girls, when compared to nonconforming boys (Coyle et al., 2016; Sullivan et al., 2018; Thomas & Blakemore, 2013). Greater disapproval for boys displaying counterstereotypical behavior is common in adult (Feinman, 1981; Martin, 1990) and even teacher (Fagot, 1977) samples. It has been theorized that the harsher reaction to male violations of stereotypes is due to society’s patriarchal values, which place masculinity above femininity in terms of status and desirability (Berger et al., 1972; Feinman, 1981; Nauts, 2015; Rudman & Glick, 2010).

### Possibility of Positive Effects for Gender Nonconformity?

Much research has focused on the pressing issue of negative backlash toward gender atypical children (Coyle et al., 2016; Sullivan et al., 2018; Thomas & Blakemore, 2013), but gender atypicality can be evaluated positively by others (Bochicchio et al., 2019; Meimoun et al., 2024). Empirical and theoretical associations between perceived prosocial behavior, self-esteem, and creativity are explored in the present study.

#### Prosocial Behavior

Prosocial behavior is defined as “voluntary actions intended to benefit another” (Skoe et al., 2002, p. 296) and tends to be stereotyped as feminine (Eisenberg et al., 2006; Quenneville et al., 2022). Evidence from educational settings showed feminine boys to be perceived as more prosocial than masculine boys by teachers (Piché & Plante, 1991). The link between femininity and prosociality has also been cited in research conducted in daycare. Young children showed no significant gender differences in observed prosocial behavior, but their childhood educators perceived girls as significantly more prosocial than boys (Bouchard et al., 2015).

#### Creativity

Creativity has been linked to artistic pursuits (Dumas & Dunbar, 2016) but can also capture the ability to think and behave in unique ways that “diverge from the normative” (Proudfoot et al., 2015). Individuals showing greater gender atypicality could be characterized as displaying eccentric behavior and behaving in ways that deviate from the norm. Empirically, emphasizing an individual’s eccentricity has been related to an increase in perceived creativity of said individual (Van Tilburg & Igou, 2014). Such associations are not surprising given cultural stereotypes of eccentric geniuses or peculiar artists; defying social norms could be seen as thinking “outside the box,” a persistent belief about creative people (Proudfoot et al., 2015; Weisberg, 2010). These results suggest that gender atypicality could be associated with being evaluated as being more creative.

#### Self-Esteem

Self-esteem refers to a person’s evaluation of themselves, with high self-esteem being a positive sense of self-regard (Zeigler-Hill & Myers, 2012). Actual rates of self-esteem in gender atypical people can be low (Egan & Perry, 2001), due in part to factors such bullying (Hu et al., 2024) and restrictive societal norms (Zentner & Von Aufsess, 2022). However, actual and perceived self-esteem can (Zeigler-Hill et al., 2013), but do not necessarily correlate (Kilianski, 2008; Watson et al., 2002). Perceived self-esteem may thus offer different insight and is worth investigating.

Greater adherence to gender typical behavior has been associated with greater felt pressure to conform to gender norms (Cook et al., 2019, p. 1913), which in turn was associated with low self-esteem (Egan & Perry, 2001; Good & Sanchez, 2010; Skinner et al., 2018). It could follow that those who display counterstereotypical behavior might have freed themselves from pressure to conform, benefiting their self-esteem. Individuals who display some gender atypicality are perhaps more likely to be ascribed greater self-esteem by others due to their disengagement from societal expectations around gender, despite facing possible backlash.

### Cultural Considerations

The available investigations into gender stereotypes of young children and reactions to violations of stereotypes by adults have been primarily conducted in the USA. It would be beneficial to establish the content of gender stereotypes for this age group in a European sample, as evidence showed that gender stereotypes can differ between German and American cultures (Wilde & Diekman, 2005). Investigations into reactions toward gender atypical children and adolescents from European countries showed differences to findings from the USA (Sullivan et al., 2018; Thomas & Blakemore, 2013), with the former finding some positive opinions regarding gender atypical individuals (Bochicchio et al., 2019; Meimoun et al., 2024). It is not established whether it is possible to extrapolate findings on gender differences from American to German or even European society.

## Sampling Considerations

While previous studies offered important insight into gender stereotypes and adult reactions to child development (Sullivan et al., 2018; Thomas & Blakemore, 2013), the adults sampled were not chosen for their familiarity with children. Parents and teachers represent relevant socializing agents from which children receive subtle or explicit feedback about gender-appropriate and inappropriate behavior, according to social cognitive theory (Bussey & Bandura, 1999). Investigating how pedagogical faculty engages with gender nonconformity is especially relevant, as they will experience interactions with a wide variety of children as compared to most adults and even parents. Specifically, we will focus on faculty in training, as they represent a new generation in the educational workforce and can give insight into whether current training is preparing teachers for fostering gender sensitivity in educational settings (Koordinationsstelle: Chance Quereinstieg/Männer in Kitas, 2019; Kultusministerkonferenz, 2016).

### Study Overview

The overall purpose of this research is thus twofold: firstly, to examine gender stereotypes about children in German society; and secondly, to investigate the impact that violations of these stereotypes, as well as masculinity and femininity, have on evaluations of children by educational professionals in training.

In order to investigate these questions, our current study was influenced by the empirical work of Sullivan et al. (2018), whose investigation ascertained descriptive, prescriptive, and proscriptive stereotypes about children in American society and tested whether violations of these stereotypes related to backlash from adults. We follow the outline of their experiments, adapting material as necessary in order to investigate stereotypes in German society and with a more specific sample of adults, namely pedagogical educators in training.

We will first investigate stereotypes about 3-year-old children in German society. Descriptive, prescriptive, and proscriptive stereotypes will be gathered in study 1 in order to collect material for vignettes, which will be used in Study 2, a procedure used in Sullivan et al. (2018). Following established procedure (Koenig, 2018; Nauts, 2015; Rudman et al., 2012b; Sullivan et al., 2018), our first study will ask adult participants to indicate the degree to which certain characteristics are considered typical or desirable for young children by German society. This should reveal descriptive, prescriptive, and proscriptive stereotypes about children in Germany.

Using the stereotypes from Study 1, we will investigate whether children adhering to or violating these stereotypes will be regarded differently by pedagogical educators in training. With our investigation, we hope to expand upon findings in past literature, using a new cultural and sampling context but keeping the established methods used previously (Sullivan et al., 2018). The past literature outlines an effect of gender atypicality on liking, finding that particularly feminine boys were more disliked than other children, and that girls tended to be rated higher in terms of liking (Sullivan et al., 2018). We hypothesize that children behaving in a nonstereotypical manner will be liked less than typical children (H1a) and that the atypical boy will receive the greatest backlash in terms of liking (H1b). We also expect girls to receive higher ratings in terms of liking than boys (H1c).

The relation between competence and gender atypicality is not straightforward. For children, findings showed that behaving atypically, enacting positive masculinity and femininity, can be perceived as rather competent, although typical girls were rated as most and “sissies” as the least competent (Coyle et al., 2016), suggesting additional research is necessary. We expect that ratings of competence will differ between children behaving in line with and in violation of gender stereotypes (H2).

In terms of internalizing and externalizing problems, gender atypicality was not a relevant factor in past research; instead, gender typing of behavior as masculine or feminine predicted externalizing and internalizing tendencies, although this past study did not use established measures (Thomas & Blakemore, 2013). We hypothesize that perceptions of internalizing and externalizing tendencies will differ between masculine and feminine children (H3a). Specifically, perceptions of internalizing problems should be higher in feminine children (H3b). Masculine children are expected to be perceived as showing greater externalizing tendencies (H3c).

Since gender atypicality may be associated with further constructs, we will also investigate prosociality, creativity, and self-esteem. Prosociality has been related to atypicality, showing atypical boys were perceived as more prosocial (Piché & Plante, 1991), likely due to the stereotyping of helping behaviors as feminine (Eisenberg et al., 2006; Quenneville et al., 2022). We hypothesize that atypical boys (H4a) and feminine children (H4b) will receive higher ratings of prosociality.

A theoretical link between gender atypicality and being evaluated as being creative and as having high self-esteem is possible due to the associations that “rejecting the norm” has in society. We hypothesize that stereotype violating children will be perceived as more creative than those who conform to gender stereotypes (H5). Similarly, children who behave in gender nonconforming ways are hypothesized to be perceived as having higher self-esteem than children who conform to gender stereotypes (H6).

## Study 1

## Method

### Participants

An a-priori power analysis was conducted to estimate the sample size needed to find effect sizes of at least │*d│* = 0.4 (Koenig, 2018; Nauts, 2015; Rudman et al. 2012b; Sullivan et al., 2018) with power of 80%. The power analysis was completed in G*Power 3 (Faul et al., 2007) and revealed that a between-participants design would require 100 participants per cell. Just shy of this goal, we recruited 397 participants from a university in a large German city to take part in this research. Participants who indicated that they did not understand the instructions or were missing more than 25% of the data were excluded from this analysis (*n* = 7). Of the remaining 390 participants, 250 identified as female, while 104 identified as male and a further 12 indicated identifying as a different gender and 24 did not indicate a gender. The mean age of the sample was 23.01 years (*SD* = 3.97, range = 18–43). A total of 71% of participants came from what would be classed as humanities, while 24% studied natural sciences, with roughly 5% of participants not indicating their studied subject.

### Materials and Procedure

A list of 72 characteristics was compiled for this study. The items were based on items from past research (Kessels, 2005; Koenig, 2018; Liben & Bigler, 2002; Sullivan et al., 2018; Tobin et al., 2010) and in certain cases modified to suit a German sample (e.g., changing “baseball” to “soccer”). Items included “plays soccer” and “dances ballet,” as well as “brave” and “helpful.”

The research took place during regular class time and was completed using paper and pencil questionnaires under supervision of the researcher. After reading a short introductory text outlining consent and the nature of the experiment, participants moved onto the experimental phase. Participants were made aware that their judgments should not be based on their own opinion of what is typical or desirable but instead should reflect what they believed to be typical or desirable “in German society.” Participants were instructed to indicate “how typical [or desirable, depending on condition] it is in German society for three-year-old boys [or girls, depending on condition] to display the following traits and behaviors.” The prompt was based on past research (Koenig, 2018; Prentice & Carranza, 2002; Rudman et al., 2012b; Sullivan et al., 2018) and modified for a German speaking sample. Participants were asked to rate these items in terms of their typicality or desirability on a nine-point Likert scale ranging from 1 (not at all [typical/desirable] for a [boy/girl]) to 9 (very [typical/desirable] for a [boy/girl]). Our 2 (target gender: male, female) × 2 (stereotype rating: descriptive, prescriptive) design was conducted between-participants.

## Results

### Analytical Plan

The method for determining descriptive and pre-/proscriptive stereotypes for girls and boys (or women and men, in the case of other research) was outlined by Rudman et al. (2012b) and adapted from Prentice and Carranza (2002) and has since been used in multiple studies (Koenig, 2018; Nauts, 2015; Sullivan et al., 2018, 2022). To attain descriptive stereotypes, in a first step, the means for each item are calculated separately for boys and girls. Independent samples *t* tests are conducted for each item, comparing the typicality rating for boys and girls. A trait is deemed a descriptive stereotype if (1) its mean is above a “6,” (2) the independent samples *t* test for ratings between girls and boys is significant, and (3) the effect size for the difference is larger than │*d│* > 0.4. All three criteria must be satisfied for a characteristic to be labeled a descriptive stereotype.

Prescriptive stereotypes are obtained through similar criteria, though these stereotypes are drawn from the scales assessing desirable traits. In order for a characteristic to be labeled a prescriptive stereotype, the mean for items must be rated above a 6 on the desirability scale, the difference between ratings for boys and girls must reach the significance threshold, and the effect size for the difference needs to be larger than │*d│* > 0.4.

Proscriptive stereotypes differ slightly in that the mean on the desirability scale must be below 4 and thus considered undesirable. Similar to descriptive and prescriptive stereotypes, the differences between ratings of girls and boys must be significant and have an effect size larger than│*d│* > 0.4.

### Analyses

We analyzed both the typicality and desirability ratings for boys and girls of 72 items. After calculating descriptive statistics, we conducted a series of independent samples* t* tests, comparing ratings for boys and girls and ascertaining Cohen’s *d* effect sizes for each *t* test. Since items were rated both on typicality and desirability, it is possible for items to be considered both descriptive, prescriptive, and proscriptive (or any combination thereof).

### Descriptive, Prescriptive, and Proscriptive Stereotypes

We found a large number of descriptive items for both boys and girls; see Table 1 for items. The items that showed the highest means and strongest effect sizes for descriptive stereotypes pertained to activities and appearance of children rather than more internal traits. We found a large number of prescriptive stereotypes for girls and boys. The items with the largest effect sizes related to appearance and play preferences, a finding also displayed by characteristics rated as desirable for boys. We found fewer proscriptive stereotypes, compared to both descriptive and prescriptive stereotypes. The most “forbidden” characteristics for boys (lowest means) were appearance related items, like wearing dresses and skirts or wearing nail polish. The least desirable traits for girls to have related to behaviors such as fighting, being loud, or playing with a wooden sword. A correlation between items showed that items measuring typicality and desirability for ratings for girls was *r* = .88, while a similar analysis for boys displayed a correlation of *r* = .89.

**Table 1:** Means, standard deviations, and Cohen’s *d* for items on the descriptive and prescriptive scales

Item	Descriptive scale			Prescriptive scale		
	Mean (SD) Girl	Mean (SD) Boy	Cohen's *d*	Mean (SD) Girl	Mean (SD) Boy	Cohen's *d*
Plays rough and tumble games	2.63 (1.30)	7.27 (1.67)	− 3.11***	2.31 (1.34)	5.56 (2.01)	− 1.90***
Plays with Playmobil™	5.52 (1.88)	6.75 (1.61)	− 0.71***	5.52 (1.62)	6.65 (1.81)	− 0.66***
Has a dollhouse	7.69 (1.46)	2.38 (1.63)	3.44***	7.34 (1.67)	2.71 (1.48)	2.94***
Builds paper planes	4.38 (1.69)	6.3 (1.71)	− 1.13***	4.77 (1.47)	7.13 (1.66)	− 1.51***
Plays with a wooden sword♂	2.67 (1.35)	7.25 (1.49)	− 3.22***	3.1 (1.52)	7.28 (1.52)	− 2.75***
Fearless	3.96 (1.68)	5.75 (1.60)	− 1.09***	4.38 (1.68)	7.04 (1.8)	− 1.53***
Wild	4.43 (1.68)	6.82 (1.42)	− 1.53***	3.17 (1.62)	6.42 (1.68)	− 1.97***
Plays soccer	3.33 (1.56)	7.58 (1.62)	− 2.68***	4.16 (1.9)	7.82 (1.49)	− 2.15***
Plays cops and robbers♂	3.93 (1.89)	7.26 (1.52)	− 1.94***	3.95 (1.9)	7.35 (1.5)	− 2.00***
Shy	6.42 (1.53)	4.62 (1.16)	1.32***	5.95 (1.7)	3.42 (1.33)	1.66***
Plays with tools	2.73 (1.49)	6.52 (1.71)	− 2.37***	3.51 (1.68)	7.05 (1.6)	− 2.16***
Tough	4.98 (1.9)	5.67 (1.65)	− 0.39***	4.59 (1.63)	6.7 (1.58)	− 1.31***
Dominant	4.27 (1.94)	5.64 (1.75)	− 0.74**	3.38 (1.75)	6.07 (1.93)	− 1.46***
Wears pink	7.48 (1.69)	2.01 (1.18)	3.74***	6.86 (1.6)	2.53 (1.38)	2.91***
Plays house	7.81 (1.63)	3.48 (1.81)	2.51***	7.16 (1.6)	4.28 (1.96)	1.60***
Has short hair	3.49 (1.57)	7.8 (1.54)	− 2.77***	3.79 (1.46)	7.29 (1.6)	− 2.29***
Rebellious	4.31 (1.65)	6.42 (1.48)	− 1.35***	3.05 (1.53)	5.64 (1.74)	− 1.58***
Collects stickers	6.45 (1.71)	4.89 (1.93)	0.85***	6.34 (1.39)	5.1 (1.69)	0.80***
Has long hair	7.72 (1.4)	3.16 (1.37)	3.29***	7.48 (1.51)	3.61 (1.54)	2.54***
Helpful	6.8 (1.44)	4.86 (1.12)	1.50***	7.94 (1.2)	6.56 (1.88)	0.87***
Brave	5.28 (1.51)	6.18 (1.41)	− 0.61***	6.08 (1.65)	7.39 (1.48)	− 0.83***
Wears blue	4.38 (1.53)	7.16 (1.52)	− 1.82***	4.67 (1.19)	6.63 (1.47)	− 1.46***
Persistent	4.77 (1.51)	6.06 (1.28)	− 0.93*	3.84 (1.62)	6.02 (1.44)	− 1.43***
Plays in a toy kitchen♀	7.48 (1.25)	3.54 (1.72)	2.63***	7.09 (1.51)	3.83 (1.65)	2.06***
Plays outside	7.02 (1.41)	7.46 (1.56)	− 0.29*	6.94 (1.67)	8.00 (1.33)	− 0.71***
Plays with cars♂	3.42 (1.67)	8.07 (1.13)	− 3.25***	3.94 (1.44)	7.7 (1.35)	− 2.70***
Plays dress-up	7.56 (1.26)	4.84 (2.05)	1.60***	7.03 (1.59)	4.87 (1.81)	1.27***
Considerate	6.55 (1.61)	4.2 (1.38)	1.56***	7.76 (1.37)	6.26 (1.94)	0.89***
Has good manners	6.53 (1.49)	4.15 (1.35)	1.68***	8.07 (1.32)	6.96 (1.69)	0.73***
Likes horses	7.45 (1.36)	3.26 (1.69)	2.74***	6.68 (1.42)	3.84 (1.64)	1.86***
Proud	5.47 (1.28)	6.06 (1.41)	− 0.44***	5.41 (1.49)	6.25 (1.62)	− 0.54***
Likes sports	5.37 (1.62)	7.43 (1.35)	− 1.38***	6.29 (1.42)	8.05 (1.00)	− 1.44***
Plays with blocks	4.51 (1.77)	7.56 (1.29)	− 1.96***	5.09 (1.31)	7.54 (1.22)	− 1.93***
Plays with dolls♀	8.08 (0.98)	2.43 (1.37)	4.77***	7.41 (1.47)	2.83 (1.45)	3.14***
Wears nail polish	6.26 (2.25)	1.75 (1.43)	2.39***	5.69 (2.26)	1.98 (1.44)	1.98***
Patient	4.96 (1.82)	3.56 (1.46)	0.85***	7.27 (1.37)	5.84 (1.92)	0.86***
Sings in a children's choir	5.31 (1.5)	3.63 (1.53)	1.11***	5.88 (1.67)	4.49 (1.47)	0.88***
Neat	6.21 (1.75)	3.19 (1.38)	1.92***	7.55 (1.51)	5.66 (1.68)	1.18***
Obedient	6.36 (1.55)	4.04 (1.31)	1.62***	7.72 (1.38)	6.01 (1.85)	1.04***
Jumps rope	6.79 (1.53)	4.11 (1.59)	1.72***	6.59 (1.42)	4.73 (1.54)	1.26***
Sensible	6.18 (1.7)	4.08 (1.22)	1.41***	7.54 (1.34)	6.01 (1.86)	0.94***
Active	6.27 (1.44)	7.63 (1.23)	− 1.01***	6.47 (1.6)	7.88 (1.14)	− 1.02***
Draws dresses♀	7.18 (1.3)	2.25 (1.41)	3.63***	6.65 (1.69)	2.77 (1.46)	2.46***
Likes princesses♀	8.07 (1.2)	2.04 (1.44)	4.55***	7.31 (1.64)	2.3 (1.27)	3.41***
Cuddly	7.04 (1.48)	4.55 (1.7)	1.57***	7.24 (1.46)	4.77 (1.76)	1.53***
Talkative	6.43 (1.39)	5.61 (1.76)	0.52***	6.44 (1.64)	6.38 (1.45)	0.04
Wears skirts and dresses	7.94 (1.22)	1.55 (1.1)	5.49***	7.34 (1.55)	1.9 (1.14)	4.00***
Goes fishing	2.13 (1.34)	5.16 (1.94)	− 1.82***	3.53 (1.65)	6.02 (1.58)	− 1.55***
Strong	3.57 (1.84)	6.33 (1.47)	− 1.66***	4.04 (1.78)	7.17 (1.46)	− 1.92***
Energetic	5.28 (1.72)	7.00 (1.31)	− 1.12***	4.4 (1.81)	6.91 (1.28)	− 1.61***
Emotional	7.38 (1.02)	4.63 (2.01)	1.74***	6.43 (1.82)	4.07 (1.8)	1.30***
Dances ballet♀	7.21 (1.54)	1.69 (1.11)	4.09***	6.77 (1.62)	2.24 (1.38)	3.01***
Cautious	6.22 (1.52)	3.65 (1.34)	1.80***	7.04 (1.4)	4.61 (1.65)	1.59***
Does horseback-riding	6.43 (2.04)	2.66 (1.57)	2.07***	6.41 (1.55)	3.42 (1.72)	1.82***
Likes dinosaurs	3.44 (1.67)	8.08 (1.08)	− 3.28***	4.24 (1.6)	7.57 (1.2)	− 2.36***
Effortful	6.52 (1.62)	4.29 (1.38)	1.48***	7.72 (1.33)	6.3 (1.75)	0.91***
Likes superheroes	3.98 (1.73)	7.89 (1.3)	− 2.55***	4.46 (1.56)	7.34 (1.35)	− 1.97***
Loving	7.00 (1.39)	4.88 (1.67)	1.38***	7.44 (1.29)	5.64 (1.67)	1.21***
Friendly	7.07 (1.29)	5.48 (1.14)	1.31***	8.22 (1.24)	7.01 (1.57)	0.86***
Plays hop-scotch	7.12 (1.46)	4.27 (1.73)	1.79***	7.02 (1.53)	4.65 (1.81)	1.42***
Climbs trees	4.99 (1.8)	7.43 (1.24)	− 1.58***	4.63 (1.73)	7.23 (1.33)	− 1.69***
Has a chemistry set	2.97 (1.87)	5.74 (1.67)	− 1.56***	3.81 (1.82)	6.24 (1.66)	− 1.39***
Recites poems	5.41 (1.93)	3.15 (1.56)	1.29***	5.99 (1.77)	4.39 (1.69)	0.93***
Does gymnastics	6.41 (1.6)	3.3 (1.66)	1.91***	6.6 (1.34)	4.29 (1.7)	1.51***
Ice-skates	5.85 (1.89)	3.92 (1.67)	1.08***	6.16 (1.48)	4.55 (1.94)	0.93***
Adventurous	4.91 (1.71)	7.48 (1.21)	− 1.74***	4.64 (1.66)	7.41 (1.32)	− 1.85***
Draws rocketships♂	2.98 (1.54)	7.41 (1.4)	− 3.00***	3.83 (1.58)	7.15 (1.48)	− 2.17***
Self-confident	5.27 (1.65)	6.53 (1.3)	− 0.85***	5.62 (1.72)	7.27 (1.58)	− 1.00***
Courageous	5.03 (1.65)	6.96 (1.33)	− 1.29***	5.29 (1.65)	7.58 (1.43)	− 1.49***
Flies a kite	4.89 (1.78)	6.89 (1.3)	− 1.28***	5.03 (1.57)	7.07 (1.31)	− 1.42***
Loud	5.06 (2.12)	7.5 (1.42)	− 1.35***	2.8 (1.83)	5.82 (1.98)	− 1.59***
Collects soccer-cards♂	2.02 (1.41)	7.51 (1.57)	− 3.68***	3.08 (1.65)	7.23 (1.5)	− 2.63***

Items with ♀ or ♂ denote these were used to construct feminine or masculine vignettes, respectively. Range 1–9. *p < .05; **p < .01; ***p < .001

In many ways, our results were in line with the previous establishment of gender stereotypes for young children in the USA. We found that (1) most stereotypes address appearance and activities rather than traits; (2) there is considerable overlap between descriptive and prescriptive stereotypes; and (3) there is a lower number of proscriptive stereotypes when compared to descriptive and prescriptive stereotypes. Our results differ from the past literature in that we found a very large number of stereotypes and that our effect sizes appear to be larger compared to past research (Sullivan et al., 2018).

Using information gathered in Study 1, we were able to construct vignettes for our second study. We chose to focus on items that belong in the category of descriptive and prescriptive for one gender and proscriptive for the other gender, as these showed the largest differences between ratings for girls and boys. We chose five items to signify masculinity and femininity, respectively. These items depict behavior relating to play activities observed in a kindergarten, such as playing with dolls or drawing a rocket ship. We will use these gender stereotyped behaviors to investigate whether children who display masculine or feminine behavior in line with their gender will be evaluated differently than children who defy gender stereotypes.

## Study 2

## Method

### Participants

We conducted an a-priori power analysis in G*Power (Faul et al., 2007) showing our study to require around 380 participants. We recruited a sample of pedagogical educators in training. In Germany, these pedagogical educators are trained to be able to work with children, adolescents, and young adults in environments such as kindergartens, care homes, and youth centers (Bundesagentur für Arbeit, 2023).The process of becoming a pedagogical educator in this federal system involves practical experience in a pedagogical environment prior to taking classes in vocational school for at least six semesters while also working in an educational institution for an extended amount of time to gain further practical experience. We recruited six vocational schools from two federal states in and around a large German city and sampled 448 of their pedagogical educators in training. The sample was 75% female, which, while being heavily skewed, is actually more equal in terms of gender representation than actual kindergarten teachers, a job dominated by female staff (93% female) (Autorengruppe Fachkräftebarometer, 2021). The remaining 23% were male, while 1% did not indicate a gender, or indicated a gender outside the male–female binary. The mean age of our sample was 27.5 (*SD* = 8.4), with a range between 18 and 59 years. The average number of years of vocational school completed by our sample was 1.7 (*SD* = 0.68), with 42% of the sample having completed their first year and a further 42% having completed their second year. On a ten-point Likert scale ranging from 0 to 9, participants rated their mean level of experience with young children as 7.4 (*SD* = 2.0).

### Materials and Design

We developed four vignettes in which a boy or a girl behaved in a masculine or feminine fashion. Two of our vignettes were thus “typical” (boy showing masculine behavior, girl showing feminine behavior), while two vignettes showed “nontypical” behavior (boy showing feminine behavior, girl showing masculine behavior). The names used in vignettes (Dominik or Linda) were chosen from a large dataset, which allowed us to match names in terms of average levels of perceived education, attractiveness, intelligence, warmth, and competence (Nett et al., 2020).

This 2 × 2 design was conducted between-participants, with the gender of the target child (male, female) and the behavior of the target child (masculine, feminine), varying between respondents. Participants thus only received one vignette, featuring a description of a masculine or feminine child, that was either a boy (Dominik) or a girl (Linda). The number of participants was roughly equal in each condition.

The vignettes, included in full in the appendix, feature a dialogue between a kindergarten teacher and a pedagogical educator in training (Natalie), who is an apprentice in the kindergarten where she gathered work experience for vocational school. Natalie describes the play activities (e.g., playing with a wooden sword and playing “cops and robbers” or playing with dolls and playing in a toy kitchen) and drawing preferences (e.g., drawing a dress or drawing a rocket ship) of a child (Dominik or Linda) with whom she is not familiar, with the fellow kindergarten teacher offering more information about the child’s preferences (e.g., liking cars or princesses) and hobbies (e.g., dances ballet or collects soccer-cards) and saying that such activities are very typical of this child.

### Measures

We constructed scales in order to measure perceived liking and competence of the child. Whether the rater liked the child was assessed with a single item measure (“How much do you like this child?”—Personal liking) rated on a seven-point Likert scale, ranging from “not at all” to “very much.” A further three items (on the same seven-point Likert scale) measured how much other children and faculty would like this child (“To what extent do you think that other children would enjoy playing with this child?”—Perceived liking by others), with this scale showing an acceptable reliability (Cronbach’s *α* = .72). Competence was rated using a three-item scale consisting of items such as “Does the child seem competent?”, similarly rated on a seven-point Likert scale, which showed acceptable reliability (Cronbach’s *α* = .76). Items were derived from the literature (Sullivan, 2018) and translated and adapted for a German sample.

We further included single items relating to perceived creativity and self-esteem of the child, with each item being rated on a seven-point Likert scale ranging from “not at all creative” to “very creative” and “not a lot of self-esteem” to “a lot of self-esteem,” respectively.

Next, we measured prosocial behavior, internalizing, and externalizing tendencies using the German version of the strength and difficulties questionnaire, the SDQ-Deu (Goodman, 1997, 2001; Klasen et al., 2000; Koglin et al., 2007; Petermann et al., 2010), which consists of 25 items and has 5 subscales (5 items each): prosocial behavior, emotional problems, conduct problems, hyperactivity, peer problems. Items are rated on a three-point Likert scale: “does not apply” (0), “partly applies” (1), and “strongly applies” (2).

The scale includes items such as “restless, overactive” (hyperactive subscale, part of externalizing problems), “often fights with other children” (conduct problems subscale, part of externalizing problems), “often unhappy, downhearted” (emotional problems subscale, part of internalizing problems), “rather solitary, tends to play alone” (peer problems subscale, part of internalizing problems), and “kind to younger children” (prosocial behavior subscale). Five items were reverse-coded in line with SDQ scoring procedures (https://www.sdqinfo.org/py/sdqinfo/c0.py), and means for subscales were calculated from relevant items. Prosocial behavior was obtained by summing scores from the relevant subscale and showed acceptable internal reliability *α* = .77. An indication of internalizing and externalizing behavior was obtained by summing the subscales peer problems and emotional problems (internalizing behavior) and the subscales hyperactivity and conduct problems (externalizing behavior). Both composite scales showed acceptable reliability (internalizing behavior Cronbach’s *α* = .77; externalizing behavior Cronbach’s *α* = .84).

### Procedure

Data were collected in vocational schools using paper questionnaires distributed by the research team. The procedure took roughly 15 minutes and took place in the classroom. After receiving a form outlining informed consent and data protection, participants gave consent verbally, in line with procedures recommended by ethical guidelines. Participants were given a paper packet containing one of the four possible vignettes (masculine girl, feminine girl, masculine boy, or feminine boy) and the questionnaires. After reading the vignette and the instruction to picture the child portrayed in the vignette, participants answered questions about their impressions of the child. First, participants indicated their personal liking of the child, followed by their impression about whether other children and educators would like the child in the vignette. Secondly, participants indicated the perceived level of competence, followed by the perceived creativity and self-esteem of the child. Participants then moved onto the SDQ-Deu, which gathered information about the ascribed internalizing, externalizing and prosocial tendencies of the target child. Finally, participants provided some demographic details about themselves before returning the questionnaires to the research team.

## Results

### Analytical Plan

After investigating the data for exclusionary criteria (such as indicating guessing the purpose of the experiment or ticking boxes in a systematic pattern), we excluded 34 cases, leaving 414 participants. Data were analyzed with SPSS 27 and SPSS 29 (IBM Corp, 2020). We checked for normality using a visual inspection of histograms and QQ plots, showing acceptable normality to use parametric inferential tests.

We calculated a series of two-way ANOVAs in order to investigate the impact of gender stereotype (masculine/feminine) and target gender (male/female) of our vignette on multiple dependent variables. As we investigated a large number of variables and the main effects of gender, gender stereotyped behavior, and the interactions of these factors, we applied corrections for multiple testing. Given that our study is one of the first studies investigating this question in this specific population, in this specific culture, we opted for a more conservative correction to mitigate the risk of type Ι errors (Anderson, 2008). We thus applied Bonferroni correction to these results, multiplying each of our *p*-values by the initial number of statistical tests carried out (24) (Anderson, 2008, p. 1485). Please note that, in these cases, it is common to receive *p*-values that exceed the value of 1 (Anderson, 2008, p. 1485).^1^ Some of our initial results reached significance levels of *p* < .001, in these cases we used a *p*-value of 0.0009 in our calculations, since a more precise indication of exact *p*-values was not provided by our statistical program. We report the original *p*-values in italics before the corrected *p*-values written in standard format, using the latter, corrected values to interpret our findings.

### Liking

Descriptive statistics can be found in Table 2, and correlations of dependent variables are presented in Table 3. In terms of personal liking for the child in the vignette, there were no significant interactions of gender and gender stereotyped behavior (*F*(1, 408) = 6.83, *p* = *0.009*/0.216, *pre*/post correction), or significant main effects of gender (*F*(1, 408) = 3.79, *p* = *0.052*/1.248) or gender stereotype (*F*(1, 408) = 0.36, *p* = *0.547*/13.128).

**Table 2:** Means and standard deviations for dependent variables

Item	Masculine boy Mean (SD)	Feminine boy Mean (SD)	Masculine girl Mean (SD)	Feminine girl Mean (SD)
Perceived liking by others^a^	4.88 (0.84)	4.53 (1.00)	4.87 (0.94)	4.83 (0.96)
Personal liking^a^	4.64 (1.03)	4.98 (1.21)	4.71 (1.09)	4.50 (0.91)
Competence^a^	5.18 (0.92)	5.37 (1.00)	5.55 (1.00)	5.00 (1.03)
Creativity^a^	5.44 (1.35)	5.74 (1.03)	5.94 (1.06)	5.24 (1.12)
Self-esteem^a^	5.63 (1.03)	5.90 (1.10)	6.21 (0.94)	4.78 (1.24)
Prosocial behavior^b^	5.04 (1.83)	7.69 (1.80)	6.25 (2.20)	6.67 (2.00)
Internalizing problems^c^	4.68 (3.26)	7.33 (3.25)	4.94 (3.27)	6.50 (3.05)
Externalizing problems^c^	9.56 (4.03)	4.68 (2.77)	7.79 (4.00)	4.41 (2.65)

^a^Range from 1 to 7; ^b^Range from 0 to 10; ^c^Range from 0 to 20

**Table 3:** Correlations for dependent variables

Variable	1	2	3	4	5	6	7	8
Liking by others	–							
Personal liking	.37**	–						
Competence	.49**	.35**	–					
Internalizing problems	− .34**	− .06	− .32**	–				
Externalizing problems	− .05	− .06	− .13*	.04	–			
Prosociality	− .05	− .09	− .13**	− .03	.37**	–		
Creativity	.26**	.30**	.47**	− .24**	− .15**	− .15**	–	
Self-esteem	.18**	.31**	.41**	− .18**	.08	− .01	.49**	–

*p < .05; **p < .01; ***p < .001

The results showed no significant interactions (*F*(1, 410) = 2.71, *p* = *.101*/2.424) or main effects of gender (*F*(1, 410) = 2.55, *p* = *.111*/0.888) and gender stereotyped behavior (*F*(1, 410) = 4.39, *p* = *.037*/2.664) for liking of children by other children or faculty (perceived liking by others). These results are not in line with our hypothesis, which stated that we would find significant differences in regard to liking (H1a), especially strong backlash for feminine boys (H1b), and increased liking for girls (H1c).

### Competence

Our two-way ANOVA revealed a significant interaction of gender and gender stereotyped behavior for ratings of competence: (*F*(1, 409) = 14.67, *p* < *0.001*/0.0216, partial *η*^2^ = .035). We investigated simple main effects (with Bonferroni correction) using SPSS syntax, revealing that the masculine girl was perceived as significantly more competent than the feminine girl (*F*(1, 409) = 16.26, *p* < .001), and the masculine boy (*F*(1, 409) = 7.21, *p* = 0.008). The feminine boy was perceived as significantly more competent than the feminine girl (*F*(1, 409) = 7.46, *p* = 0.007), while the masculine and feminine boys did not differ significantly from one another (*F*(1, 409) = 1.93,* p* = 0.166). We expected significant differences between children behaving in stereotypical and nonstereotypical ways in regards to ratings of competence (H2) and found partial support for this hypothesis in the case of the masculine girl (*M* = 5.55, *SD* = 1.0), who was perceived as more competent than the feminine girl (*M* = 5.00, *SD* = 1.03); in contrast, gender stereotyped behavior was not related to differences in competence for the feminine (*M* = 5.37, *SD* = 1.0) and masculine boys (*M* = 5.18, *SD* = 0.92).

### Strength and Difficulties Questionnaire

#### Internalizing and Externalizing Behavior

Our two-way ANOVA revealed that there was no significant interaction between child gender and gender stereotyped behavior (*F*(1, 407) = 2.98, *p* = *0.085*/2.04) for internalizing behavior. Results showed no significant main effect of child gender (*F*(1, 407) = 0.80, *p* = *0.371*/8.904). However, we saw a significant main effect of stereotyped behavior: *F*(1, 407) = 44.16, *p* < *0.001*/0.0216, partial η^2^ = .098, showing that the feminine girl (*M* = 6.50, *SD* = 3.05) and boy (*M* = 7.33, *SD* = 3.25) were given greater internalizing scores than the masculine girl (*M* = 4.94, *SD* = 3.27) and boy (*M* = 4.68, *SD* = 3.26). This result supports our hypothesis H3b, which stated that we would find a difference in internalizing behavior, with feminine children (*M* = 6.91, *SD* = 3.17) being rated as having higher scores than masculine children (*M* = 4.81, *SD* = 3.26).

From inferential analyses, we did not have a significant interaction between child gender and gender stereotyped behavior regarding externalizing problems (*F*(1, 407) = 4.91, *p* = *0.027*/0.648). While we had no significant main effect of child gender (*F*(1, 407) = 9.04, *p* = *0.003*/0.072), we see significant main effects of stereotyped behavior (*F*(1, 407) = 148.17, *p* < *0.001*/0.0216, partial *η*^2^ = .267). The masculine boy (*M* = 9.56, *SD* = 4.03) and girl (*M* = 7.79, *SD* = 4.00) were perceived as having more externalizing tendencies than the feminine boy (*M* = 4.68, *SD* = 2.77) and girl (*M* = 4.41, *SD* = 2.65), lending support to our hypothesis H3c, which stated that masculine children (*M* = 8.68, *SD* = 4.10) would be ascribed greater externalizing problems than feminine children (*M* = 4.54, *SD* = 2.71). As both internalizing and externalizing subscales differed between masculine and feminine children, our hypothesis H3a was also fully supported.

#### Prosocial Behavior

Analyses revealed a significant interaction between child gender and gender stereotyped behavior, *F*(1, 406) = 33.27, *p* < *0.001*/0.0216, partial *η*^2^ = .076. Simple main effects analyses were conducted and revealed that the feminine boy was perceived as significantly more prosocial than the masculine boy *F*(1, 406) = 93.31, *p* < .001. The masculine boy, in turn, was seen as less prosocial than the masculine girl, *F*(1, 406) = 19.86, *p* < .001. The feminine boy was also seen as more prosocial than the feminine girl, *F*(1, 406) = 13.70, *p* < .001. For girls, gender stereotyped behavior did not make a significant difference in perceived prosocial behavior, *F*(1, 406) = 2.28, *p* = .132. Our hypotheses stated that we expect feminine boys (H4a) and feminine children (H4b) to have favorable ratings on prosociality. We found a significant interaction between gender and gender stereotyped behavior, showing that the feminine boy was perceived as the most prosocial (*M* = 7.69, *SD* = 1.80), more so than the masculine boy (*M* = 5.04, *SD* = 1.83), supporting our hypothesis H4a, but since the masculine (*M* = 6.25, *SD* = 2.20) and feminine girls (*M* = 6.67, *SD* = 2.00) did not differ significantly from one another, hypothesis H4b was not supported.

### Creativity

Inferentially, we saw a statistically significant interaction between gender and stereotyped behavior for creativity: *F*(1, 408) = 19.35, *p* < *0.001*/0.0216, partial *η*^2^ = .045. An analysis of the simple main effects showed significant differences between the children. We saw the masculine girl judged as more creative than the feminine girl (*F*(1, 408) = 19.09, *p* < .001). The masculine girl was rated as significantly more creative than the masculine boy (*F*(1, 408) = 9.84, *p* = 0.002) and the feminine boy as significantly more creative than the feminine girl (*F*(1, 408) = 9.52, *p* = .002). We noted that there was no difference in creativity for boys, whether they were masculine or feminine (*F*(1, 408) = 3.43, *p* = .065). In our hypothesis H5, we stated that violations of gender stereotyped behavior would lead to children being ascribed greater creativity compared to children who act in accordance with gender stereotypes. We found partial support for this hypothesis, as the masculine girl (*M* = 5.94, *SD* = 1.06) was rated as more creative than her feminine counterpart (*M* = 5.24, *SD* = 1.12), while there were no significant differences in perceived creativity between the feminine (*M* = 5.74, *SD* = 1.03) and masculine boys (*M* = 5.44, *SD* = 1.35).

### Self-Esteem

The two-way ANOVA revealed a statistically significant interaction of child gender and gender stereotyped behavior, *F*(1, 409) = 64.16, *p* < *.001*/0.0216, partial *η*^2^ = .136. Simple main effects analyses revealed that the masculine girl was seen as having the highest self-esteem. She was rated as having significantly higher self-esteem than the feminine girl (*F*(1, 409) = 91.38, *p* < .001). Results showed that the feminine boy had significantly higher self-esteem than the feminine girl (*F*(1, 409) = 54.91, *p* < .001). The masculine girl was rated as having significantly higher self-esteem than the masculine boy (*F*(1, 409) = 15.12, *p* < .001). We did not see significant differences in the masculine or feminine boys in terms of self-esteem (*F*(1, 409) = 3.17, *p* = 0.076). We hypothesized that children not acting in accordance with gender stereotyped behavior for their gender would receive higher ratings on perceived self-esteem and our findings partially supported this hypothesis (H6), as girls who adhered to stereotypical behavior (*M* = 4.78, *SD* = 1.24) were perceived as having lower self-esteem than girls who acted in a masculine gender stereotyped manner (*M* = 6.21, *SD* = 0.94). The feminine (*M* = 5.90, *SD* = 1.10) and masculine boys (*M* = 5.63, *SD* = 1.03), however, did not show significant differences from one another in regards to perceived self-esteem.

## Discussion

The aim of this study was to investigate how pedagogical educators in training evaluated vignettes describing gender stereotypical and nonstereotypical three-year-old children, so as to understand whether there are systematic differences in how children are evaluated based on their gender and gendered behavior.

Our analyses showed no effect of gender or stereotyped behavior on either measure of liking. We saw an impact of gender stereotype and gender for ratings of competence, creativity, and self-esteem, which all showed that the masculine girl received higher ratings, while the feminine girl received the lowest ratings. Gender stereotyped behavior seemed not to make a large difference for boys for these variables. Interestingly, prosocial behavior showed an interaction of gender and gender stereotype, with the feminine boy perceived as most prosocial, while the masculine boy was rated as the least prosocial. Regarding the SDQ-Deu, we saw masculine children perceived as more externalizing, while feminine children were rated as having more internalizing tendencies. Gender stereotyped behavior was a significant factor here, while child gender, or nonconformity, was not. Overall, we see that femininity in boys is not viewed overwhelmingly negatively in this sample. Instead, we see negative outcomes for the feminine girl and positive outcomes for the masculine girl, specifically for ascribed competence, creativity and self-esteem.

## General Discussion

The present work investigated two main questions: which descriptive, prescriptive, and proscriptive stereotypes exist about three-year-old children in German society, and how do pedagogical educators in training evaluate children who conform to or defy these gender stereotypes? We found that adults in Germany identified gender stereotypes for three-year-old boys and girls and that these stereotypes were comparable to past research from the USA (Koenig, 2018; Sullivan et al., 2018, 2022).

In a similar vein to Koenig (2018), the typical characteristics of boys and girls showed more emphasis on activities rather than traits. Compared to girls, whose play activities heavily revolved around humanoid, caring play (dolls, princesses, playing house), boys’ activities featured more “things” (cars, dinosaurs). In terms of highest means, prescriptive stereotypes for girls were related to traits and behaviors, such as being helpful, friendly, and having good manners. These reflect communal values ascribed to women (Hentschel et al., 2019; Hsu et al., 2021). The active, physical nature of prescriptive stereotypes for boys is in line with the beliefs that “sport” is a male domain (Messner, 2011; Plaza et al., 2017). Whereas girls’ prescriptions related to their being, prescriptions for boys related to what they are doing. However, these results should be interpreted cautiously: while the top prescriptions for girls related to having good manners, the data showed that these traits were also highly desirable for boys.

Noticeably, the most proscriptive or “forbidden” characteristics for boys, in terms of lowest means, were appearance related items that signal femininity (wearing dresses and skirts, wearing nail polish, or dancing ballet). The least desirable characteristic for girls related to aggressive behaviors, such as fighting, or being loud. Negative masculinity is partly captured by violent and aggressive tendencies (Krahé et al., 2007), which seem represented in the items deemed proscriptive for girls. Proscriptive items for boys related to feminine physical appearance and clothing. It thus seems that *femininity*, removed from positive and negative labels, was proscriptive for boys. This finding is in line with the theoretical argument stating that prescriptive and proscriptive stereotypes serve to maintain hierarchical systems (Nauts, 2015; Rudman & Glick, 2010). This positive evaluation of masculinity is found in the results of study 2. Masculine girls received the most flattering ratings in terms of competence, creativity, and self-esteem, while feminine boys were not evaluated differently than masculine boys on these measures.

### Liking

Prior research has shown that nontypical children were liked less than their typical peers (Sullivan et al., 2018). Despite some acceptance of gender atypicality for “tomboys” and “mama’s boys,” gender typical girls were viewed as most likable in past research, with boys showing negative femininity rated as the least likable (Coyle et al., 2016). Contrary to our hypotheses, we found no effect on liking, which perhaps is linked to our sample: pedagogical faculty are discouraged from showing personal preferences. Overall, our present study found no differential treatment between children adhering to or violating gender stereotypes in terms of “liking.”

### Competence

Previously, typical girls were rated as the most and “sissies” rated as the least competent, while “mama’s boys” and “tomboys” were rather positively evaluated as possessing higher competence than the typical boy (Coyle et al., 2016). For adolescents, a gender atypical target was seen as competent, but only by participants with high SES (Meimoun et al., 2024). Our results stand in contrast with some past findings, showing significant impact of gender nonconformity on ratings of competence for girls but not boys. For ratings of competence, masculine and feminine boys were perceived similarly. As masculinity and androgyny have been associated with competence (Heilbrun, 1981; Korlat et al., 2022; Martin & Slepian, 2021), our finding that masculine girls benefitted from greater masculinity is in line with this literature. We believe that the counterstereotypical nature of masculinity in girls may account for the significant difference we see between masculine girls and boys. As masculinity in girls is unexpected, it might therefore be evaluated as more extreme and given more credence than masculinity displayed by a boy (cf. Streck et al., 2022).

### Internalizing and Externalizing Problems

Past research showed that rather than nonconformity, it was the gender typing of the child that predicted internalizing and externalizing tendencies (Thomas & Blakemore, 2013). Our current study expanded upon these findings using the established SDQ, which has previously been used as a measure of such adjustment problems (Goodman et al., 2010). We found a similar pattern as Thomas and Blakemore (2003), showing that masculine children were ascribed greater externalizing problems, while feminine children were ascribed greater internalizing tendencies. We did not find an effect of gender but only of gender stereotyped behavior, showing that it is not necessarily conformity to gender but expressions of gender that relate to perceived problems.

### Prosocial Behavior

In our findings on average, feminine children were rated as more prosocial than masculine children. A closer look revealed that the feminine boy was rated as the most prosocial, significantly more so than the masculine boy and feminine girl. Finding feminine boys to be considered more prosocial than masculine boys has also been shown in children previously (Piché & Plante, 1991). Notably, the feminine and masculine girls did not differ significantly from one another in terms of prosociality. There is evidence showing childhood educators believed girls to be more prosocial than boys, despite no observed differences in prosociality between genders (Bouchard et al., 2015). Perhaps the girlhood of the masculine girl activated the “female” stereotype of prosociality (Eisenberg et al., 2006; Quenneville et al., 2022), enough to outweigh her masculine tendencies.

### Creativity

Our analyses showed significant interactions of gender and gender stereotyped behavior in terms of perceived creativity. Overall, the masculine girl received beneficial ratings on creativity, while gender stereotyped behavior was not a significant factor for boys. Eccentricity has been linked to greater perceived creativity (Van Tilburg & Igou, 2014), although this would not account for our gender specific findings. The greater creativity ascribed to masculine girls could be seen as an endorsement of the connection between creativity and masculinity (Proudfoot et al., 2015). The masculine girl may have benefitted from the association between creativity and both atypicality and masculinity, leading to the finding that she was seen as the most creative.

### Self-Esteem

In regard to perceived self-esteem of children adhering to or violating gender stereotypes, we found a similar pattern as for creativity and competence, with the greatest self-esteem ascribed to the masculine girl and the lowest self-esteem ascribed to the feminine girl, while gender stereotyped behavior was not a significant factor for boys. People behaving in ways that are seen as outside the norm are perceived as brave innovators, not bound by social convention—their self-esteem is not impacted by the opinions of others. Our results are in line with literature linking actual, not perceived, self-esteem to psychological androgyny (Bem, 1974) and masculinity or instrumentality (Antill & Cunningham, 1979; Heilbrun, 1981; Marsh et al., 1987; Whitley & Gridley, 1993), which showed that specifically women can benefit from more masculine orientations (Heilbrun, 1981; Streck et al., 2022; Whitley, 1988). This would also explain why the feminine boy was not seen as having particularly high self-esteem, despite disregarding social norms: his femininity is not conducive to high self-esteem, unlike high masculinity, which is strongly related to positive self-esteem (Antill & Cunningham, 1979; Whitley & Gridley, 1993). We note, however, that perceived self-esteem does not necessarily relate to actual self-esteem (Kilianski, 2008; Watson et al., 2002).

### Implications

Past research has revealed a consistent and strong bias against feminine boys, who received the greatest backlash. The focus on the negative aspect of femininity in boys did not emerge in our findings. Interestingly, femininity in our study was only “punished” in girls, while boys displaying the same behavior did not receive backlash in terms of competence, creativity, and self-esteem. Indeed, the feminine boy was perceived as the most prosocial, suggesting that femininity can be an asset for boys but a liability in girls. Our results support the notion that femininity is seen as “lesser than” masculinity. Gender can be seen as a status characteristic, with masculinity having higher status than femininity (Berger et al., 1972; Feinman, 1981). Denigration of the feminine begins as early as kindergarten, with feminine activities given less time and space than masculine activities (Prioletta & Davies, 2022). Denigrating patterns (Hill & Augoustinos, 1997) continue in school, where feminine achievement has been attributed to effort, rather than ability (Espinoza et al., 2014; Fennema et al., 1990). Whereas younger girls often preferred “pink, frilly dresses” (Halim et al., 2014), girls in elementary school girls reported greater affinity for a tomboy aesthetic (Halim et al., 2011). This effect was theorized to relate to increased understanding of status of gender by girls as they age. From a status perspective it is not surprising that a girl would want to enact masculinity, this, in fact, supports the existing gender hierarchy, as it shows that masculinity is desirable. A boy deigning to engage with femininity, however, threatens the hierarchy by casting high status masculinity aside and undermining its value (Feinman, 1981). This violation is then punished more harshly in order to sustain the hierarchical gender system. While the lack of masculinity in boys was viewed critically in past studies (Feinman, 1981; Sullivan et al., 2018; Thomas & Blakemore, 2013), the presence of it was rewarded for girls in our present results. Greater masculinity in girls could be implicitly encouraged by pedagogical educators in training or in wider society. There is evidence to suggest that women have become increasingly masculinized in the past decades (Twenge, 1997; Wilde & Diekman, 2005), yet not all investigations yield similar patterns (Eagly et al., 2020; Haines et al., 2016). Changing gender stereotypes (Eagly et al., 2020) may reflect a shift in views of masculinity and femininity.

Sanctioning boys who defy gender stereotypes could be seen as a response to a world in which such individuals experience worse outcomes than their stereotypical counterparts (Folkierska-Żukowska et al., 2022; Hu et al., 2024; Issler et al., 2023). Encouraging girls to take on masculine traits could also be interpreted as awareness of the benefits associated with this gender role orientation. Studies showed that instrumentality can be a beneficial resource for adolescent girls in academic contexts (Streck et al., 2022), and that the association between agency or masculinity and self-esteem was particularly strong for women (Hirokawa & Dohi, 2007; Streck et al., 2022; Whitley, 1988). Of course, by displaying differential reactions to gender stereotyped behavior, even if to attempt to protect boys from poorer future outcomes or encourage beneficial outcomes in girls, socializing agents are perpetuating the power of stereotypes and hierarchy of gender. Encouraging boys and girls to embrace both masculinity and femininity, free from backlash, could have considerable benefits in academic contexts, where androgyny and gender atypical orientations have been linked to higher achievement, self-esteem, and school-related well-being (Korlat et al., 2022; Yavorsky & Buchmann, 2019; Yu et al., 2020) and for constructing a less gender stereotyped society as a whole (Bem, 1981b).

### Limitations and Future Research Directions

Ideally, we would have liked to include a measure of social desirability in order to indicate whether participants may have been providing answers in line with norms of social equity. Future studies may wish to add items measuring social desirability bias, a suggestion laid out previously (Sullivan et al., 2018). Collecting further demographic details of participants, such as their SES, could also be advantageous in future, as past research showed that high SES individuals displayed different result patterns than low SES individuals for attitudes toward gender atypicality (Meimoun et al., 2024). Similarly, investigating whether the gender of the evaluating party has an impact on results would be interesting. Due to our overwhelmingly female sample, we would not have sufficient power to detect an effect of participant gender. Past research typically has not found this factor to have a significant impact on most variables (Coyle et al., 2016; Sullivan et al., 2018; Thomas & Blakemore, 2013).

Whether our findings could generalize to children of other ages is uncertain. Past research has shown that multiple stereotypes of 3- and 4-year-old children were also applied to 7-year-old children, supporting the idea that stereotypes can generalize across age groups (Sullivan et al., 2018, 2022). The theoretical link between atypicality and perceived creativity and self-esteem should apply to other age groups.

We would also state that further expansion of this research could include sampling kindergarten teachers who have passed their examinations. Perhaps there is a difference between pedagogical educators in training and kindergarten teachers working full-time. Examining how experienced teachers react to vignettes of nonconformity, or perhaps even instances of actual nonconformity, such as in past research (Fagot, 1977), could benefit the literature and offer a more naturalistic methodology. We chose to work with vignettes, as this enabled a systematic comparison across conditions, while manipulating only gender and gender stereotyped behavior. We used a between-participants design, rather than a within-participants design, as presenting multiple vignettes to participants could have made our experimental manipulation—and thus our hypotheses—obvious to participants and influenced their responses (Charness et al., 2012).

Future studies should investigate whether opinions expressed by pedagogical educators in training are in line with intended behavior toward gender nonconforming children. While we assessed approval and disapproval by measuring constructs like liking and competence, we have no indication of whether these reactions toward gender nonconforming children will translate into behavioral intentions. Italian pre-service teachers claimed they would adopt a supportive, rather than corrective, stance toward children showing gender nonstereotypical behavior (Bochicchio et al., 2019). Combining the behavioral intent from this investigation with the more affective focus of our study would offer interesting insight.

Comparing our results to past research, while insightful, also comes with a major caveat, as we conducted our research in a different culture to most past research and chose a highly specific sample. While studies conducted in the USA (Coyle et al., 2016; Sullivan et al., 2018; Thomas & Blakemore, 2013) found results of backlash, investigations originating in Italy and France revealed generally more positive attitudes toward gender atypicality from pre-service teachers (Bochicchio et al., 2019) and adolescents (Meimoun et al., 2024). Along with our findings this calls for more cross-cultural research into perceptions of gender atypicality.

We believe this research, informed by social cognitive theory, applies specifically to enactive experience and direct tuition, as these modes of social influence require feedback from a social agent, in this case pedagogical educators in training. The role of adults in the development of gender typed behaviors and attitudes is explored not just in social cognitive theory, but also in gender schema theory (Martin & Halverson, 1981). According to this theory, socializing agents can serve to label certain activities, behaviors, and materials (clothes, toys) as being gender-appropriate or inappropriate, which can then inform a child’s gender schema, shaping their future cognitions and behaviors. Experimentally labeling novel toys as “for girls” or “for boys” impacted how much children were interested in or enjoyed a particular toy (Weisgram et al., 2014), while gender inappropriate toys were cast aside like a “hot potato” (Martin et al., 1995, p. 1467). Even the use of novel colors as gender labels can impact children, who reported greater liking of gender-appropriate colors (Yeung & Wong, 2018). These findings underscore the relevance of cues from adult socializers for children’s attitudes and behaviors.

### Conclusion

The results outline that gender stereotypes are a present factor in the lives of adults and children. Pedagogical educators in training reported an interesting pattern, showing a preference for masculinity over femininity in girls—a pattern worth investigating further. More efforts need to be made in order to achieve the goal of gender equity in the German educational system, as outlined by German political actors (Kultusministerkonferenz, 2016) and the wishes of German parents (Wößmann et al., 2018).

## Data Availability

Available upon request and exclusively in line with guidelines set by the Senate Department for Education, Youth, and Family of our federal state.

## English Vignettes (Translated from German)

### Introduction

In the following, you will read a short story set in a Kindergarten.

Try to put yourself into the situation and picture the child from the story. Afterwards we will ask you to please answer some questions about the child.

Today was Natalie’s first day as a pre-service educator in the Kindergarten where she previously completed an internship. In the kitchen she runs into a Kindergarten teacher, who has been working at the Kindergarten for a while.

### Feminine Girl

**Kindergarten teacher:** Oh, hello Natalie! How was everything on your first day? Did everything go well with the children?

**Natalie:** Good, thanks! Overall, it went very well! I already know a lot of the kids from my internship here, and they know me too. The only one I didn’t know was Linda, one of the three-year-olds. What is she like? Today on the playground, she played with dolls the whole time. And then she wanted to play in the toy kitchen. When we were drawing later, she drew a dress.

**Kindergarten teacher**: Yes, that is very typical. Linda also likes princesses and likes to dance ballet.

### Feminine Boy

**Kindergarten teacher**: Oh, hello Natalie! How was everything on your first day? Did everything go well with the children?

**Natalie:** Good, thanks! Overall, it went very well! I already know a lot of the kids from my internship here, and they know me too. The only one I didn’t know was Dominik, one of the three-year-olds. What is he like? Today on the playground, he played with dolls the whole time. And then he wanted to play in the toy kitchen. When we were drawing later, he drew a dress.

**Kindergarten teacher:** Yes, that is very typical. Dominik also likes princesses and likes to dance ballet.

### Masculine Girl

**Kindergarten teacher**: Oh, hello Natalie! How was everything on your first day? Did everything go well with the children?

**Natalie:** Good, thanks! Overall, it went very well! I already know a lot of the kids from my internship here, and they know me too. The only one I didn’t know was Linda, one of the three-year-olds. What is she like? Today on the playground, she played with a wooden sword the whole time. And then she wanted to play “cops and robbers.” When we were drawing later, she drew a rocket ship.

**Kindergarten teacher:** Yes, that is very typical. Linda also plays with cars a lot and likes showing her soccer-cards.

### Masculine Boy

**Kindergarten teacher:** Oh, hello Natalie! How was everything on your first day? Did everything go well with the children?

**Natalie**: Good, thanks! Overall, it went very well! I already know a lot of the kids from my internship here, and they know me too. The only one I didn’t know was Dominik, one of the three-year-olds. What is he like? Today on the playground, he played with a wooden sword the whole time. And then he wanted to play “cops and robbers.” When we were drawing later, he drew a rocket ship.

**Kindergarten teacher:** Yes, that is very typical. Dominik also plays with cars a lot and likes showing his soccer cards.

## Footnotes

1 In our analysis, a pre-correction *p*-value of .05 would be multiplied by 24 (the number of tests we carried out), yielding a post-correction *p*-value of 1.2. Ordinarily, a non-corrected *p*-value could only range between 0 and 1, yet the multiplicative correction makes *p*-values exceeding 1 possible and likely (Anderson, 2008, p. 1485). As the post-correction value (1.2) is far above the accepted significance threshold of .05, we would reject this hypothesis. While some of our pre-correction results may seem significant, our post-correction results show non-significance.

## Code Availability

Available upon request.
